# Generation of iPS Cells from Human Hair Follice Dermal Papilla Cells

**Published:** 2014

**Authors:** I. A. Muchkaeva, E. B. Dashinimaev, A. S. Artyuhov, E. P. Myagkova, E. A. Vorotelyak, Y. Y. Yegorov, K. S. Vishnyakova, I. E. Kravchenko, P. M. Chumakov, V. V. Terskikh, A. V. Vasiliev

**Affiliations:** Koltsov Institute of Developmental Biology, Russian Academy of Sciences, Vavilova str., 26, 117808, Moscow, Russia; Pirogov Russian National Research Medical University, Ostrovitianov str., 1, 117997, Moscow, Russia; Engelhardt Institute of Molecular Biology, Russian Academy of Sciences, Vaviolva str., 32, 119991, Moscow, Russia; Shemyakin and Ovchinnikov Instituse of Bioorganic Chemistry, Russian Academy of Sciences, Miklukho-Maklay str., 16/10, 117997, Moscow, Russia

**Keywords:** Hair follicle dermal papilla (DP) cells, induced pluripotent stem (iPS) cells, reprogramming

## Abstract

Dermal papilla (DP) cells are unique regional stem cells of the skin that induce formation of a hair follicle and its regeneration cycle. DP are multipotent stem cells; therefore we supposed that the efficiency of DPC reprogramming could exceed that of dermal fibroblasts reprogramming. We generated induced pluripotent stem cells from human DP cells using lentiviral transfection with Oct4, Sox2, Klf4, and c-Myc, and cultivation of cells both in a medium supplemented with valproic acid and at a physiological level of oxygen (5%). The efficiency of DP cells reprogramming was ~0.03%, while the efficiency of dermal fibroblast reprogramming under the same conditions was ~0.01%. Therefore, we demonstrated the suitability of DP cells as an alternative source of iPS cells.

## INTRODUCTION

The generation of induced pluripotent stem (iPS) cells , which are of significant interest in medicine and developmental biology, is one of the topical problems of stem cell research. Advanced cell technologies allow one to generate various iPS cell lines that can be used to model diseases and test new drugs. Developing iPSC-based therapy is associated with the generation of products for replacement enzyme therapy and, further, for cell transplantology.

Hair follicle dermal papilla cells are regional stem cells of the skin and a unique subject for study. They play the key role in the morphogenesis of a hair follicle and its growth cycle. Reorganization of hair is a result of a series of inductive interactions between skin epithelial and mesenchymal cells during the growth cycle of a hair follicle. Back in 1967, Oliver demonstrated that DP cells can induce the generation of hair follicles [1]. DP cells of scalp hair originate from the neural crest; DP cells of the dorsal side of the body are derivatives of the somite dermatome, while DP cells of the visceral body side originate from the splanchnotom visceral layer. It is well known that the WNT , BMP, and FGF signal paths act in intact DP cells [2]; their activation promotes maintenance of the inductive properties of cultured DP cells. The most commonly tested markers of dermal papilla include alkaline phosphatase (AP), versican, CD133 (in mice), Noggin, and Lef1. ALP is probably the most accurate marker of DP cells. Maximum expression of this enzyme is observed during early anagen. ALP is considered to be an indicator of the DP induction potential. Versican is an extracellular matrix proteoglycan synthesized during the anagen. A number of proteins, including the aforementioned ones, very often cannot be detected during DP cell passage, and the induction potential of DP cells disappears with time.

By the time we started our study, no data or publications devoted to the reprogramming of DP cells of the human hair follicle were available. A number of authors have assumed that successful reprogramming of somatic cells to a pluripotent state is impossible without induced expression of the four pluripotency transcription factors Oct4, Sox2, Klf4, and c-Myc [3-5]. Higgins* et al*. [6] demonstrated that Klf4 and c-Myc begin to be expressed in cultured cells, while they cannot be detected in intact DP. In contrast, another reprogramming factor, Sox2, is synthesized in intact DP but not in culture [7]. However, two years earlier, in 2010, the same authors had denied the fact that Sox2 synthesizes in human DP cells [6]. With allowance for these facts, we set out to use DP cells as an alternative source of iPS cells and to analyze their reprogramming efficiency. We assumed that at least two (Klf4 and c-Myc), or even three (Sox2, Klf4, and c-Myc), reprogramming factors may be synthesized in non-reprogrammed DP cells, this promoting cell transition to the pluripotent state. The cells were cultured in a medium supplemented with valproic acid [8] and at a physiological oxygen level (5%) [9] to increase reprogramming efficiency. Therefore, our study was aimed at generating iPS cells from human DP cells under set conditions and at comprehensively characterizing the generated iDP cells.

## EXPERIMENTAL

**Cell cultures**

The following cell cultures were used in the study: human hair follicle dermal papilla (DPC) cells; hESMK05 human embryonic stem cells, kindly provided by M.A. Lagarkova (Vavilov Institute of General Genetics, Russian Academy of Sciences), and primary mouse embryonic fibroblasts (MEFs).

DP cells were isolated according to our own procedure [10] and cultured in AmnioMAX II (Gibco) supplemented with penicillin (50 U/ml) and streptomycin (50 µg/ml) (Paneco). The medium was changed every 3–4 days. As soon as a cell layer reached the subconfluent state, the cells were passed at a 1 : 3 ratio. hESMK05 and reprogrammed iPS cells were cultured in mTeSR™ 1 (STE MCE LL Technologies) at 37°C, 5% CO2 and 5% O2. PSCs were passed either mechanically, with a plastic tipped pipette, or by incubation in a 1 mg/ml dispase solution (STE MCE LL Technologies) on a cultural substrate Matrigel, a mixture of extracellular matrix proteins and proteoglycans (BD Biosciences); the medium was changed daily.

**Generation of iDP cell clones**

DP cells of the third passage (0.5 mln per a 30–60 mm Petri dish) were infected with lentiviral vectors carrying the pluripotency genes (*Oct4, Sox2, Klf4*, and *c-Myc*) in the DP cells culture medium supplemented with 5 µg/ ml Polybrene (Sigma) at 37°C, 5% CO_2_, and 5% O_2_ overnight. Through the first seven days, the medium was supplemented with small molecule compounds enhancing reprogramming efficiency, 2 mM valproic acid (Sigma) and 50 µg/mL ascorbic acid (Sigma), and changed every other day. On day 8, the reprogramming medium was replaced with the medium for pluripotent stem cells, mTeSR™ 1 (STE MCE LL Technologies), and was further changed daily. Cells in their first passages were mechanically plated onto mitotically inactive MEFs and, with the beginning of colony contacting, onto Matrigel-coated plastic dishes (BD Biosciences). The aforementioned additives were discontinued from the first cell passage onto 100 mm Petri dishes. Within 2–3 weeks after passing, iPSC clones were selected according to a PSC-surface marker, Tra-1-60, using vital immunolabeling and mechanical selection of positive clones.

**Alkaline phosphatase activity assay**

The activity of AP was assayed using an NBT/BCID kit (Roche), according to the manufacturer’s protocol.

**Immunocytochemistry and flow cytometry**

For immunofluorescent assay, the cells were fixed in a 4% paraformaldehyde solution. The cells were incubated with primary antibodies (see the Table) in PBS with either 1% BSA (Helicon) or 5% FBS (BioWest) at 4°C overnight, and then with the secondary antibodies, Alexa Fluor 488, Alexa Fluor 594, or Texas Red, at the ambient temperature for 60 min. Nuclei were counterstained with 4,6-diamidino-2-phenylindole (DAPI) in PBS (Vector Laboratories) at the ambient temperature for 10 minutes. The specimens were analyzed on an Olympus IX51 fluorescent microscope (Olympus) and a Coolpix 8700 digital camera (Nicon). Cells expressing PSC surface antigens were quantified on a Cell Lab Quanta™ SC flow cytometer (Beckman Coulter). Three replicates of a sample, at least 30,000 cells each, were analyzed.

**Telomerase activity test**

Cell telomerase activity was assayed using the TR APEZE ® XL Telomerase Detection Kit (Millipore) and Encyclo polymerase (Evrogen) as a PCR enzyme.

**RT-PCR gene expression assay**

Total RN A was isolated with the use of Quick-RN A™ MiniPrep (Zymo Research) according to the manufacturer’s protocol, slightly modified. Reverse transcription (RT ) was performed using the RT -PCR kit and oligo(dT)15-primers (Sileks). Primers were selected according to Ohnuki *et al*. [11] from the primer bank http://pga.mgh.harvard.edu/primerbank/index.html. PCR was performed using a ScreenMix-HX (Evrogen) and a Bio-Rad amplifier. PCR products were separated by electrophoresis in 2% agarose gel in a Tris acetate buffer and visualized using a UV gel analyzer (BioRad).

**Real-time PCR gene expression assay**

Real-time PCR was performed using HS-SYBR (Evrogen), and cDNA generated with reverse transcription was analyzed as follows: cDNA (from 15 ng of RN A) and 10 µg of each primer selected from the primer banks http://pga.mgh.harvard.edu/primerbank/index. html and http://medgen. ugent.be/rtprimerdb were used for each reaction; cDNAs diluted 4-, 16-, 64-, and 256-fold in three replicates were used to build a calibration curve. Amplification was performed according to the following program: 95°C – 10 min, followed by 40 two-stage cycles: 95°C – 15 s, 60°C – 1 min in a 7500 Real-Time PCR System (Applied Biosystems). A melting profile of the reaction products was constructed after 40 cycles and used to analyze reaction specificity and by-products.

**Generation, culturing, and analysis of embryoid bodies (EBs)**

EBs were generated by culturing in either a AggreWell ™ (STE MCE LL Technologies) or DMEM/F12 (Gibco) medium supplemented with a 20% KSR serum substitute (Gibco); 1 × NE AA (Gibco); 1 × GlutaMAX (Gibco); penicillin (50 U/ml), and streptomycin (50 µg/ml, Paneco). Each embryoid body was formed in a hanging drop and grown in the suspended state in Ultra Low Adhesion Plates (Corning), preventing cellplastic attachment. The medium was changed daily. EBs were fixed in 3% paraformaldehyde, incubated in a 15% sucrose solution, embedded into Tissue-Tek® OCT ™ Compound (Sakura)σ and frozen in liquid nitrogen. Next, 7.5 µm thick sections were cut with a cryotome (CM1900) and layered onto positively charged microscope slides. Non-specific binding was blocked in PBS with a 1% BSA solution. Further, the samples were prepared as aforesaid.

**Directed *in vitro *iPSC differentiation**

Both pieces of embryoid bodies and undifferentiated cells (clusters occupying 10–20% of the cultural dish surface) were used for differentiation. Immunocytochemical staining was used to identify differentiation. Osteogenic differentiation was induced by cell culturing in a medium containing DMEM/F12 (Gibco), 10% FBS (Gibco), 2 mM glutamine (Paneco), 10 ng/ml hepatocyte growth factor (HGF, Invitrogen), 10 ng/mL epidermal growth factor (EGF, PeproTech), 20 ng/ ml bone morphogenetic protein 2 (BMP2, R&D), and 0.03 mM nicotinamide. In case of support-grown cells forming ESC-type colonies, the entodermal differentiation program was induced by supplementing the medium with activin A (R&D) at a 100 ng/ml concentration during the first 2–3 days (no activin A was required for differentiation from EBs). After discontinuation of activin A, the medium was supplemented with 30 ng/ ml of fibroblast growth factor 4 (FGF4, Invitrogen). As soon as the cell layers reached 80% confluence, FGF4 and BMP2 were removed from the medium and the cells were further cultivated with oncostatin M (10 ng/ ml, R&D) and dexamethasone (0.1 µM, Sigma-Aldrich) for 5–7 days. After passaging, 1 Χ B27 (Gibco) was added to the medium for the next 7–10 days. To induce neural differentiation, 20 ng/mL of both Noggin (Invitrogen) and fibroblast growth factor 2 (FGF2, Pepro- Tech) were added to DMEM/F12 (Gibco) with 3% FBS (Gibco), 1 Χ × NE AA (Gibco), 1 Χ GlutaMAX (Gibco), penicillin (50 U/ml), and streptomycin (50 µg/ml) (Paneco) for seven days. The cells were then cultivated with 1 Χ B27 (Gibco); seven days later, 10 ng/ml of both the neurotrophic growth factor (BDNF, PeproTech) and nerve growth factor β (NGFβ, PeproTech) were added and the cells were cultivated for the next 7–14 days.

**Karyotyping**

To prepare chromosome plates, colcemid (Colcemid, Gibco) was added to the culture medium. After colcemid incubation, the cells were kept in a hypotonic solution (60 mM KCl) and fixed in methanol: ice acetic acid (3 : 1) cooled to –20°C. The resulting suspension was dropped onto microscopic slides from a height of approximately 10 cm and the fixator was fired on the glass. For the differentiated staining, the chromosome plates were processed with fluorescent dyes DAPI and 7-aminoactinomycin D (7-AAD).

**Teratoma formation test**

The pluripotent state of induced cells was assayed using the standard *in vivo *teratoma formation test. Approximately 5•10^6^ undifferentiated cells were injected subcutaneously to Nude immunodeficient mice. After tumor formation, the mouse was euthanized; the tumor was cut out and fixed; histologic and immunohistologic samples were prepared.

**List of used antibodies:** 

Antibodies (antigens)	Manufacturer	Antibody producer	Working dilution
Oct4	Millipore (MAB4401)	Mouse	1 : 200
Sox2	Cell Signalling (#3579)	Rabbit	1 : 250
Nanog	Abcam (ab80892)	Rabbit	1 : 400
SSEA3	STEMCELL Technologies (#01553)	Rat	1 : 100
SSEA4	Abcam (ab16286)	Mouse	1 : 250
Tra-1-60	Abcam (ab16288)	Mouse	1 : 100
Tra-1-81	STEMCELL Technologies(#01556)	Mouse	1 : 300
Desmin	Abcam (ab15200)	Rabbit	1 : 200
Nestin	Millipore (MAB5326)	Mouse	1 : 200
Doublecortin	Abcam (ab77450)	Rabbit	1 : 200
β-III-tubulin	Millipore (MAB1637)	Mouse	1 : 100
Neuron-specific enolase	DakoCytomation (M087329)	Mouse	1 : 200
Vimentin	Abcam (ab8978)	Mouse	1 : 200
Pan-cytokeratin	Santa-Cruz (sc-81714)	Mouse	1 : 20 - 1 : 50
Osteopontin	Millipore (MAB3057)	Rat	1 : 350
Osteonectin	Millipore (AB1858)	Rabbit	1 : 500
Hnf4α	Santa-Cruz (sc-8987)	Rabbit	1 : 50
Foxa2	Millipore (#07-633)	Mouse	1 : 100
Alpha-fetoprotein	R & D Systems (MAB1368)	Mouse	1 : 200
Albumin	R & D Systems (MAB1455)	Mouse	1 : 250
CK8	Millipore (04-588)	Rabbit	1 : 200
CK18	Millipore (MAB3234)	Mouse	1 : 100
Alexa Fluor 488	Molecular Probes (# A-11029, A-21206, A-21208)	Goat, donkey	1 : 500
Alexa Fluor 594	Molecular Probes (# A-11032)	Goat	1 : 500

## RESULTS AND DISCUSSION

iPS and ES cells share a number of morphological and metabolic parameters. The study compares the generated iPS and human ES cells (hESMK05).

**Fig. 1:**
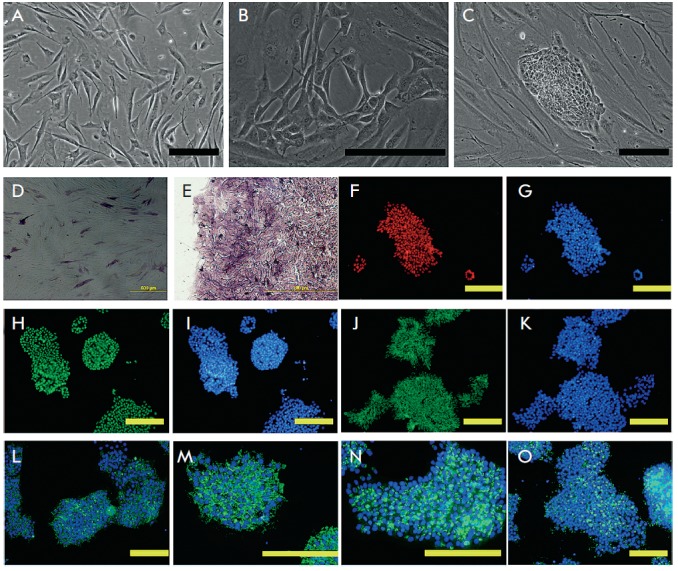
Generation of human iDP cells. Morphology of cultured DP cells before reprogramming factors infection (A) and emergence of first reprogrammed clones on the 8^th^ day after infection (B) and 11^th^ day after infection (C). Expression of alkaline phosphatase in nonreprogrammed (passage 6) DP cells (D) and in generated (passage 6) iDP cells (E). Immunofluorescent detection of nucleus proteins: Oct4(F), Sox2 (G), and Nanog (H), and surface antigens: SSEA3 (L), SSEA4(M), Tra- 1 -60 (N), and Tra -1-81 (O) in iDP cells. G, I, K, L-O - nuclei are counterstained with DAPI (blue). Scale bars = 200 μm. (G, I, K – the same visual fiels as F, H, J respectively)

**Transfection, isolation, and cultivation of iDP cells**

The early stages of reprogramming include changes in cell dimensions and shape, as well as organization of the cytoskeleton and receptors. The emergence of cells visually distinct from the initial culture can be a signal of the onset of a reprogramming process in the transfected cells. We used a histone deacetylase inhibitor, valproic acid [8], along with incubation of the cells in 5% oxygen to enhance reprogramming efficiency. We observed the emergence of the first cell colonies with an altered morphology on the 8^th^ day after lentiviral transfection of DP cell culture (*Fig*. *1B*), which points to one of the reprogramming parameters, mesenchymeepithelial transition [12]. At this moment, the reprogramming medium was replaced with mTesr1 to maintain the undifferentiated state. The colonies increased in size and new colonies emerged with time. Virtually all colonies had an ESC-like morphology and distinct shapes. The morphological parameters of all the generated clones were virtually identical. The high nucleus- to-cytoplasm ratio was typical of the cells forming these colonies (same as that for ESCs) (*Fig*. *1C*).

Only a small number of morphologically similar transformed colonies travel all through reprogramming to the pluripotent state; therefore, discriminating between the totally and partially reprogrammed clones became our next challenge. The morphological similarity of the separated clones to PSCs is a common parameter used to separate reprogrammed cells from the pool of non-reprogrammed ones. AP is one of the pluripotent state markers; hence, assay of its expression is the common first stage leading to further cloning of AP positive colonies [13, 14]. However, expression of AP is typical of intact DP cells [15], and the positive signal raised a question as to whether the AP expression resulted from cell reprogramming or from the integrity of the maternal cells parameters. Therefore, we considered selection of the clones according to this parameter to be unreasonable. To simplify the separation of totally reprogrammed iPS cells, we performed vital immunolabeling against the surface antigen Tra-1-60, one of the markers of pluripotent cells [16], on the 21^st^ day after transfection with the reprogramming factors, and then we manually selected Tra-1-60- positive colonies. We preferred the Tra-1-60 staining bearing two facts in mind: that reprogramming is a multistage process, and that this marker emerges at the later stages of acquiring pluripotency. The colonies were transferred onto a cultural plastic coated with a feeder layer of mitotically inactivated MEFs. The cells were subsequently passed onto the Matrigel-coated plastic.

**Verification of the undifferentiated state of the generated iPS cells**

AP, a marker of pluripotent and stem cells, is expressed in intact DP cells and indicates the induction capacity of DP. However, a number of proteins typical of intact DP cells, including AP, become virtually undetectable with time, and DP cells lose their induction potential [17]. Researchers consider the loss of own parameters and acquisition of interfollicular fibroblast signs a result of the absence of the microenvironment that is native to a natural DP cell culture. We detected AP expression in the isolated clones of the reprogrammed cells at the sixth passage (three passages before transfection with the *Oct4, Sox2, Klf4, *and *c-Myc *and three passages after transfection) (*Fig. 1E*), while only single cells of the initial DP culture expressed AP at the same passage (*Fig. 1D). *AP expression mainly attests to the reprogramming process taking place in cells but not always shows the totally reprogrammed state. We noticed the formation of colonies positive towards this marker, which did not become genuine iPS cells. They either could fail to totally reprogram or were rapidly undifferentiated after becoming pluripotent (and we did not manage to register this moment).

The isolated colonies were AP positive during continuous cultivation, as well. With the use of immucytochemical staining, we found that the examined clones of reprogrammed cells expressed the pluripotency transcription factors Oct4, Sox2, and Nanog (*Fig. 1 F–K*), along with the surface antigens SSEA-3, SSEA- 4, Tra-1-60, and Tra-1-81 (*Fig. 1 L–O*). We selected one iDP cell clone positive towards all the listed markers, for further study.

**Fig. 2:**
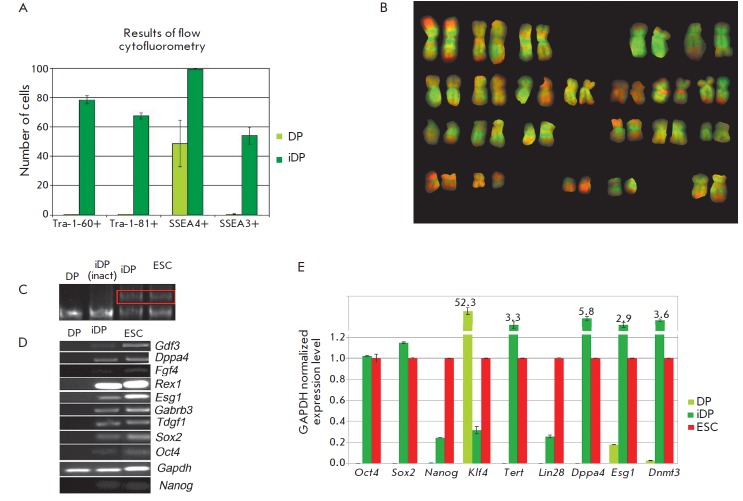
Ratio of untreated (DP) to reprogrammed (iDP) cells expressing pluripotency markers, *Tra-1 -60 , Tra-1-81 , SSEA3*, and *SSEA4*, according to flow cytofluorometry (A). Normal diploid karyotype (46,XX) of iDP cells. Chromosomes are stained with DAPI (green) and 7-AAD (red) (B). Telomerase reactivation in iDP cells (C). Telomerase is active in the samples, with additional bands corresponding to telomeric repeats (iDP, ESCs). Control: ES and iDP cells heated before telomerase inactivation (iDP) (inact). Telomerase of the initial iDP cell culture is inactive. Reverse transcription- PCR (RT-PCR) analysis of iDP cells (D). Expression levels of *Oct4, Sox2, Nanog, Tert, Klf4, Lin28, Dppa4, Dnmt3 *in DP, iDP, and ES cells (E), according to real-time PCR

However, the results of immunocytochemical staining do not show the quantitative parameters of the generated iDP. Therefore, we used flow cytometry to determine the iPS cell percentage in a culture expressing the above-mentioned surface pluripotency markers (*Fig*. *2A*). Virtually all iDP cells (99.28%) were SSEA4- positive; 78.36% of iDP cells expressed Tra-1-60; 67.39% expressed Tra-1-81, and more than half of the iDP cell population (53.94%) were SSEA3-positive. The data show that not all cells of the clone express the pluripotency antigens SSEA3, SSEA4, Tra-1-60, and Tra-1-81; however, the share of these cells is considerable (53– 99%). The selected iDP cells express SSEA3, SSEA4, Tra-1-60, and Tra-1-81, which remain virtually undetected in the initial DP cells. The maternal cells do not express these pluripotency markers, and the generated iDP cells are positive towards them; therefore, it is clear that these cells undergo reprogramming.

The average doubling time of the iDP cell population determined using the least square method was 25 h. Hence, the growth kinetics of the generated iDP was similar to that of ES cell clones (the average doubling time of the ESC population was 25 h). iDP cells of the fifth passage had normal 46,XX diploid karyotype (*Fig. 2B*).

Telomerase reactivation is another criterion of cell reprogramming to pluripotency. It is common knowledge that telomerase is inactive in somatic cells, while activated in the course of reprogramming. Telomerase acts as the reverse transcriptase elongating telomeric fragments of cell DNA. The TR AP-PCR analysis showed that telomerase of the generated iDP cells is active (*Fig. 2C*), which is not surprising considering the high proliferative potential of the cells under examination. This enzyme is inactive in the initial DP culture; therefore, iDP cell telomerase was reactivated during cell reprogramming. Telomerase activity in the generated iDP cells was similar to that in ESCs.

Using RT -PCR , we detected transcripts of the genes (*Nanog*, *Oct4*, *Sox2*, *Tdgf1*, *Gabrb3*, *Esg1*, *Rex1*, *Fgf4*,* Dppa4*, and *Gdf3*) in iPSC cultures, which are active during the early development and serve as PSC markers (*Fig. 2D*). Transcripts of these genes could not be detected in the initial DP culture; therefore, the emergence of positive signals in iDP cells indicates their activation in the course of reprogramming. Most likely, Oct4, Sox2, and Nanog activate other genes of early development. Our results confirm the key role of the above-mentioned factors in reprogramming.

We used real-time PCR to detect specific details of the culture (*Fig. 2E*) and succeeded in detecting some details in the examined cultures. However, the expression levels of virtually all the examined genes were similar in iDP and ES cells, while distinct from the expression profile in non-reprogrammed cells.

Therefore, the generated reprogrammed cells meet the criteria of undifferentiated state according to the analyzed parameters. Their further characterization required determining their differentiation potential.

**Determination of iDP cell differentiation potential**

**Fig. 3:**
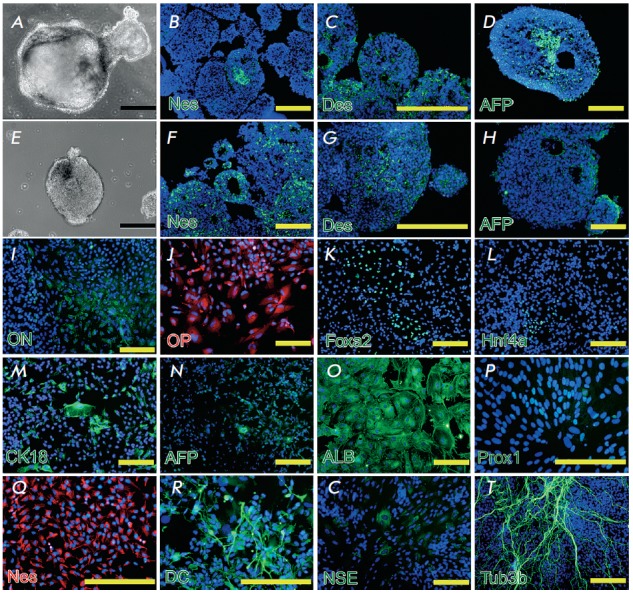
iDP cells differentiation in vitro. Formation and characterization of embryoid bodies (EBs) in iDP cell cultures (A–D), and in the control, human ES cells (E–H). Morphology of EBs in iDP (A) and ES (E) cell cultures. Expression of markers of three germinal layers, nestin (Nes), desmin (Des), and alpha-fetoprotein (AFP), EBs differentiation in iDP- (B–D) and hESderived cells (F–H) EBs. Directed differentiation of iDP cells (I–T). Expression of osteogenic differentiation markers: osteonectin (ON, I) and osteopontin (OP, J). Expression of hepatocyte differentiation markers: Foxa2 (K), Hnf4α (L), cytokeratin18 (CK18, M), alphafetoprotein (AFP, N), and albumin (ALB, O). Expression of neuronal differentiation markers Prox1 (P), nestin (Nes, Q) doublecortin (DC, R), neuron-specific enolase (NSE, S), and β-III- tubulin (Tub3b, T). Scale bars = 200 μm

We used iDP cells to generate embryoid bodies, which are similar to the early stages of embryogenesis in a laboratory environment (*Fig. 3A*). EB are self-organizing round aggregates with an internal cavity. We detected ectodermal (nestin), mesodermal (desmin), and entodermal (alphafetoprotein, AFP) proteins both in the iDP- and ESC-generated EBs (*Figs. 3 B-D*).

PSCs can be used to examine early development processes and directed differentiation of cells into a particular tissue due to their ability to differentiate under laboratory conditions. We determined the conditions for iPS cell differentiation into the ecto- (neuronal differentiation), meso- (osteogenic differentiation), and entodermal (hepatic differentiation) directions.

**Osteogenic differentiation**

Two or three weeks after induction of osteogenic differentiation of iDP cells, we detected extracellular matrix proteins, osteopontin (*Fig. 3J*) and osteonectin (*Fig. 3I*), which are regarded as osteoblast markers and participate in bone mineralization.

**Hepatic differentiation**

Hepatic differentiation was performed during 3–5 weeks; the onset of the expression of nuclear transcription factors, Foxa2 (a marker of the definitive entoderm) and Hnf4α (a marker of extraembryonic entoderm) in iDP cells, was observed within this period. Cells positive for these factors were located by groups, approximately 50–100 cells each (*Fig. 3 K, L*). Expression of two proteins emerged: alphafetoprotein (AFP) that points to hepatoblast committing of the cells and epithelial marker cytokeratine 18 (CK18), indicating a higher differentiated state of the cells (*Fig. 3 M,N*). After the differentiation, albumin expression, typical of mature hepatocytes, begins in the cells (*Fig. 3O*).

**Neuronal differentiation**

Neuronal differentiation was performed during 3–5 weeks. Immunocytochemical staining showed that neuronal differentiated iPD cells expressed Prox1 (*Fig. 3P*), which may play the pivotal role in CN S development, participating in the regulation of the expression and development of post-mitotic undifferentiated neuron precursors. Neuronal iDP cells were positive for nestin, a marker of neural stem cells, (NSC) (*Fig. 3Q*) and doublecortin (DC), a marker of immature neurons (*Fig. 3R*); they also formed plexus of cells with long processes positive towards βIII tubulin, a marker of mature neurons (*Fig. 3T*), and were positively stained towards neuron-specific enolase (NSE) (*Fig. 3S*).

***In vivo *differentiation of iPSCs**

**Fig. 4:**
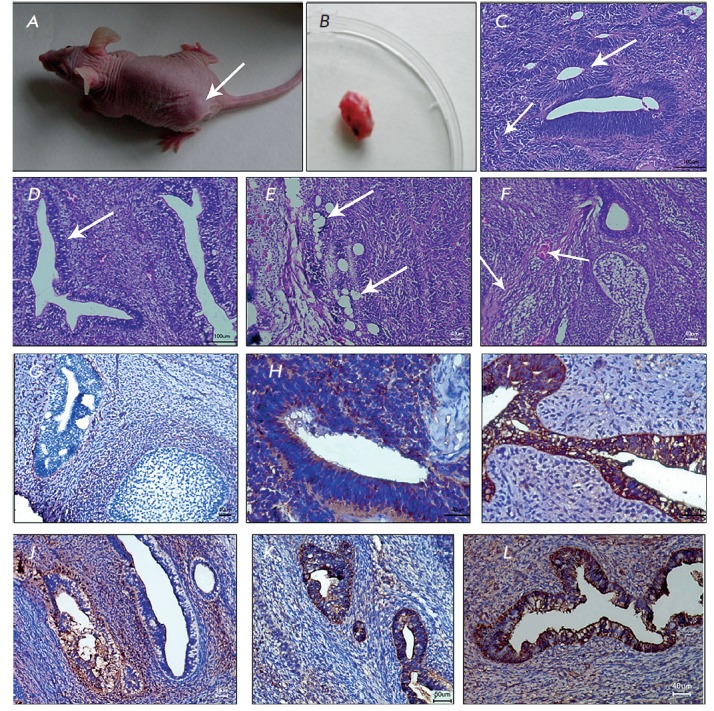
Formation of teratoma from iDP cells. The recipient mouse (A) with teratoma (arrow) and the resected teratoma (B) six weeks after subcutaneous injection of iDP cells. Histological sections of hematoxylin and eosinstained teratomas (C–F): neuroglia (neuroepithelial tubules and rosettes, arrows) (C); glandular epithelium (D), mesenchyme (adipose cells, arrows in D; fibers of loose connective tissue and vessels, arrows in F) (E, F); stained with antibodies against: vimentin (G), nestin (H) , pan CK (I) CK8 (J), CK18 (K) and AFP (L)

The ability of cells to form teratoma in thymus-free mice is one of the reliable tests that confirm their pluripotency. Teratoma is a tumor consisting of several types of tissues, usually derivatives of three germinal layers, not typical of organs or anatomical areas where the tumor develops. Data on the efficiency of teratoma formation with respect to a site of undifferentiated PSCs injection are rather ambiguous. Thus, Prokhorova *et al*. [18] reported the site-specificity of teratomas formed from the same cells, while other authors found no difference between the efficiency of the teratoma test at injection of PSCs into different areas of the recipient’s body [19]. We chose the least laborious technique of iDP cell subcutaneous injection into the back and posterior limb of a mouse. Within 3–6 weeks after the injection of undifferentiated iDP cells, a teratoma started to form at the injection site (*Fig. 4 A,B*). Histologically, this was immature teratoma, containing structures of ecto-, meso-, and entoderm origin. The mesenchymal tissues observed in histological specimens had abundant capillaries, with expanded lumens in some of them, and fibers of loose connective tissue between them (*Fig. 4F*). Moreover, it contained large cells with foam cytoplasm, a potential indicator of adipose tissue forming in the teratoma (*Fig. 4E*). Vast areas of glandular epithelium (*Fig. 4D*), along with fragments of immature nerve tissue, consisting of small hyperchromatic cells with a narrow cytoplasm rim could be observed in the specimen. Structures resembling primitive neuroectodermal rosettes and tubules were found, as well (*Fig. 4C*). The authenticity of teratoma generation from iDP cells was confirmed by immunohistochemical analysis. Vimentin-rich sites located within inter-canal tissues were detected in the specimens (*Fig. 4G*). Nestin was found in structures resembling neuroectodermal rosettes and tubules (*Fig. 4H*). We observed a large number of glandular structures in which pan-cytokeratin, cytokeratins 8 and 18, along with alphafetoprotein, were expressed (*Figs. 4 I–L*).

The reprogramming efficiency reported in [6] was ~0.02% vs. ~0.03% in our study. Most probably, this difference is insignificant and is related to the different techniques used in different laboratories. However, the result of our study might point to the positive effect of culturing conditions (low oxygen content and valproic acid supplementation) on the efficiency of reprogramming; this is quite possible, because Higgins *et al*. [6] did not mention the use of these components in their experiments. Thus, the results of these two studies are comparable and complement each other. Considering the neural crest origin of the used DP cells, further study of the generated iDP cell line, including its epigenetic parameters and tendencies towards spontaneous *in vitro *neuronal differentiation, comparatively with iPS cell lines originated from other sources is of interest.

## Abbreviations

AP: alkaline phosphatase
BSA: bovine serum albumin
DMEM/F12: Dulbecco’s Modified Eagle Medium: Ham’s Nutrient Mixture F-12
DP: dermal papilla
ES cells (ESCs): embryonic stem cells
FBS: fetal bovine serum
iDP cells (iDPCs): iPS from DP cells
iPS cells (iPSCs): induced pluripotent stem cells
PBS: phosphate buffered saline
FPA: paraformaldehyde,
PSCs: pluripotent stem cells.
